# First description of a musculoskeletal linkage in an adipose fin: innovations for active control in a primitively passive appendage

**DOI:** 10.1098/rspb.2012.2159

**Published:** 2013-01-07

**Authors:** Thomas A. Stewart, Melina E. Hale

**Affiliations:** Department of Organismal Biology and Anatomy, University of Chicago, Chicago, IL 60637, USA

**Keywords:** adipose fin, evolutionary innovation, swimming, catfish

## Abstract

Adipose fins are enigmatic appendages found between the dorsal and caudal fins of some teleostean fishes. Long thought to be vestigial, degenerate second dorsal fins, remnants of the primitive gnathostome condition, adipose fins have since been recognized as novel morphologies. Unique among the fins of extant fishes, adipose fins have uniformly been described as passive structures, with no associated musculature. Here we provide the first description of a musculoskeletal linkage in an adipose fin, identified in the sun catfish *Horabagrus brachysoma*. Modified supracarinalis posterior muscles insert from the dorsal midline anterior to the adipose fin by tendons onto the fin base. An additional pair of posterior adipose-fin muscles also inserts upon the fin base and lay posterolateral to the fin, superficial to the axial muscle. This musculoskeletal linkage is an evolutionary innovation, a novel mechanism for controlling adipose-fin movement. These muscles appear to exemplify two approaches by which fins evolve to be actively controlled. We hypothesize that the anterior muscles arose through co-option of an existing fin linkage, while the posterior muscles originated as de novo fin muscles. These findings present adipose fins as a rich system within which to explore the evolution of novel vertebrate appendages.

## 1. Introduction

The evolution of vertebrate morphology has involved repeated innovations of new musculoskeletal linkage systems. Understanding these transformations demands hypotheses of the homology of the constituent parts, the phylogenetic order of acquisition of these parts, and the selective forces that might have promoted morphological and thus developmental as well as functional change. The evolution of such innovations has been repeatedly observed in the fins of fishes, which originate as rudimentary structures and seem to evolve following a general pattern of increasing morphological and functional complexity [1]. It has been proposed that the earliest fins originated as simple, dermal projections and subsequently evolved internal skeletal supports [1]. As fins evolved greater morphological complexity, muscles arose or were co-opted, providing the potential for active control of movement [2]. The ability to control fins independently of axial musculature allowed for new axes of diversification, increased morphological disparity and functional diversity.

Scenarios of fin and limb evolution are necessarily founded upon fossils and phylogeny, but these data are inevitably limited to patterns of change among skeletal hard tissues. Surveys of the diversity of soft-tissue anatomy in extant lineages can complement fossil data and inform the repeated and general pattern of anatomical and functional elaboration in new vertebrate fins [3,4]. However, among extant fishes the diversity of fins is heavily biased towards elaborated musculoskeletal systems that permit active control of movement [5]. The origin of novel fin modules is rare and fin number is a diagnostic character for major vertebrate clades, suggesting that fin systems are heavily constrained. For example, fishes, and indeed all jawed vertebrates, are limited to at most two sets of paired appendages. Among ray-finned fishes (Actinopterygii, including teleosts) the diversity of dorsal fins observed has derived from a single fin module [6]. Adipose fins represent an important exception; situated between the dorsal and caudal fins of many teleostean fishes, they are novel structures. They have originated at least twice in actinopterygian phylogeny, once in the Otophysi clade, excluding Cypriniformes, and again in the Euteleostei, excluding *Lepidogalaxias salamandroides* [7,8]. Adipose fins are the most recent fins to evolve de novo in fishes rather than by the subdivision of an existing fin domain [6] and are generally considered to be simpler in their anatomy and function than other fins [7,9,10].

Adipose fins are morphologically distinct from other fins by several measures. The fins of nearly all actinopterygian fishes are composed of a distal dermal skeleton (fin rays and actinotrichia) and proximal endoskeletal radials [2,11]. Adipose fins contain dermal elements, which are variable and can include fin rays and actinotrichia, but in the vast majority of cases lack an endoskeleton [7]. In the few exceptions, cartilaginous plates develop at the base of the adipose fin [9]. These neomorphic endoskeletal components are proposed to be homologous to radials, because they resemble the plates in embryonic fins from which radials are known to develop [9,12]. The adipose fin endoskeleton has been described only in fishes belonging to the Euteleostei, and not in adipose fins that originated within the Otophysi.

Classically, adipose fins have been further distinguished from other fins by their lack of associated musculature [5]. Accordingly, adipose fins are hypothesized to function passively [13]. Proposed functions include affecting pre-caudal flow, serving as a sensory structure or as a mechanism for interspecific signalling [13,14]. Despite this prevailing view, some species of callichthyids, a lineage of South American armoured catfishes, have been described as having a ‘movable’ adipose-fin spine [15–20]. Regrettably, there are no descriptions of the associated anatomy; discussions are limited to a single sentence identifying ‘two strong muscle bundles’ beneath the armour of *Callichthys callichthys* that might be associated with the fin [21]. A subsequent survey of teleostean musculature that included callichthyds did not identify these muscles [22].

The rudimentary composition of adipose fins when compared with other vertebrate fins is general and not the result of the reduction of formerly elaborated fins [7]. And despite their uniqueness as recently evolved and rudimentary fins, adipose fins remain unexplored for their potential to inform the process by which novel vertebrate fins originate and evolve. Here we describe a musculoskeletal system in the adipose fin of the Asian sun catfish, *Horabagrus brachysoma.* Through dissection and histology, we identify two pairs of muscles that insert upon the fin. These muscles are a derived, specialized condition representing a new, gained functional potential in this appendage. We propose that these muscles control adipose fin position, the first description of such a mechanism in an adipose fin. These results inform general patterns of morphological and functional elaboration in novel and primitively rudimentary vertebrate appendages.

## 2. Material and methods

Research was conducted at the University of Chicago from July 2011 to April 2012, and in compliance with University of Chicago IACUC and in adherence with all legal requirements of the United States. *Horabagrus brachysoma* (*n* = 20) were obtained through the pet trade and housed at University of Chicago. Specimens ranged in size from 4.0 to 6.5 cm standard length (measured from snout to base of the caudal fin rays) and were euthanized with MS222 at a concentration of 0.5 g l^–1^. Specimens and histological slides have been donated to the Field Museum of Natural History (Chicago, IL, USA) under catalogue no. FMNH 121444.

Anatomy was characterized by dissection (*n* = 14), the serial sectioning of adipose fins and associated tissues (*n* = 4), clearing and staining (*n* = 1), and antibody staining (*n* = 1). A Leica MZ10 microscope was used for dissection and a Leica DMIRB was used to image histological slides (Leica Microsystems, Wetzlar, Germany). Photos were taken on both microscopes with an Olympus DP72 camera using cellSens Entry v. 1.2 (Build 7533) software (Olympus Corporation, Tokyo, Japan). Tissue used for sectioning was first preserved in 10 per cent paraformaldehyde for 6 days and then transferred to 70 per cent EtOH for storage. Prior to sectioning, tissue was decalcified by immersion in solution of 10 per cent EDTA and 90 per cent distilled H_2_O at pH 7.4 until lepidotrichia were flexible, approximately 3–4 days at 4°C. Tissues were paraffin embedded, sectioned at 5 μm thickness, and stained with haematoxylin and eosin by the University of Chicago's Human Tissue Resource Center (http://htrc.uchicago.edu/home.shtml). Clearing and staining followed methods adopted from Potthoff [23]. Muscles were imaged using antibody staining methods adopted from Thorsen & Hale [24], using the primary and secondary antibodies, mouse monoclonal anti-actin (α-sarcomeric; Sigma A2172) and FITC conjugated goat anti-mouse (Jackson ImmunoResearch 115-096-003), respectively. Antibody stained specimens were imaged using a Zeiss LSM 710 confocal microscope (Carl Zeiss Inc., Thornwood, NY, USA)

To explore potential muscle function, one specimen was dissected immediately following euthanasia to expose adipose fin-associated tendons. These tendons were manipulated with forceps, simulating unilateral contractions of the supracarinalis posterior (SCAR-P) muscle, while the fish was immersed in water. The resulting adipose-fin kinematics were recorded at 15 frames per second from the dorsal perspective using the above described Leica MZ10 microscope and camera. The resulting adipose-fin displacement was quantified using ImageJ [25]. An angle of rotation (θ) was calculated by measuring the displacement of the tip of the adipose fin relative to the posterior-most part of the adipose fin base.

## 3. Results

### (a) Supracarinalis posterior muscles attach to the adipose fin

**Figure 1.**
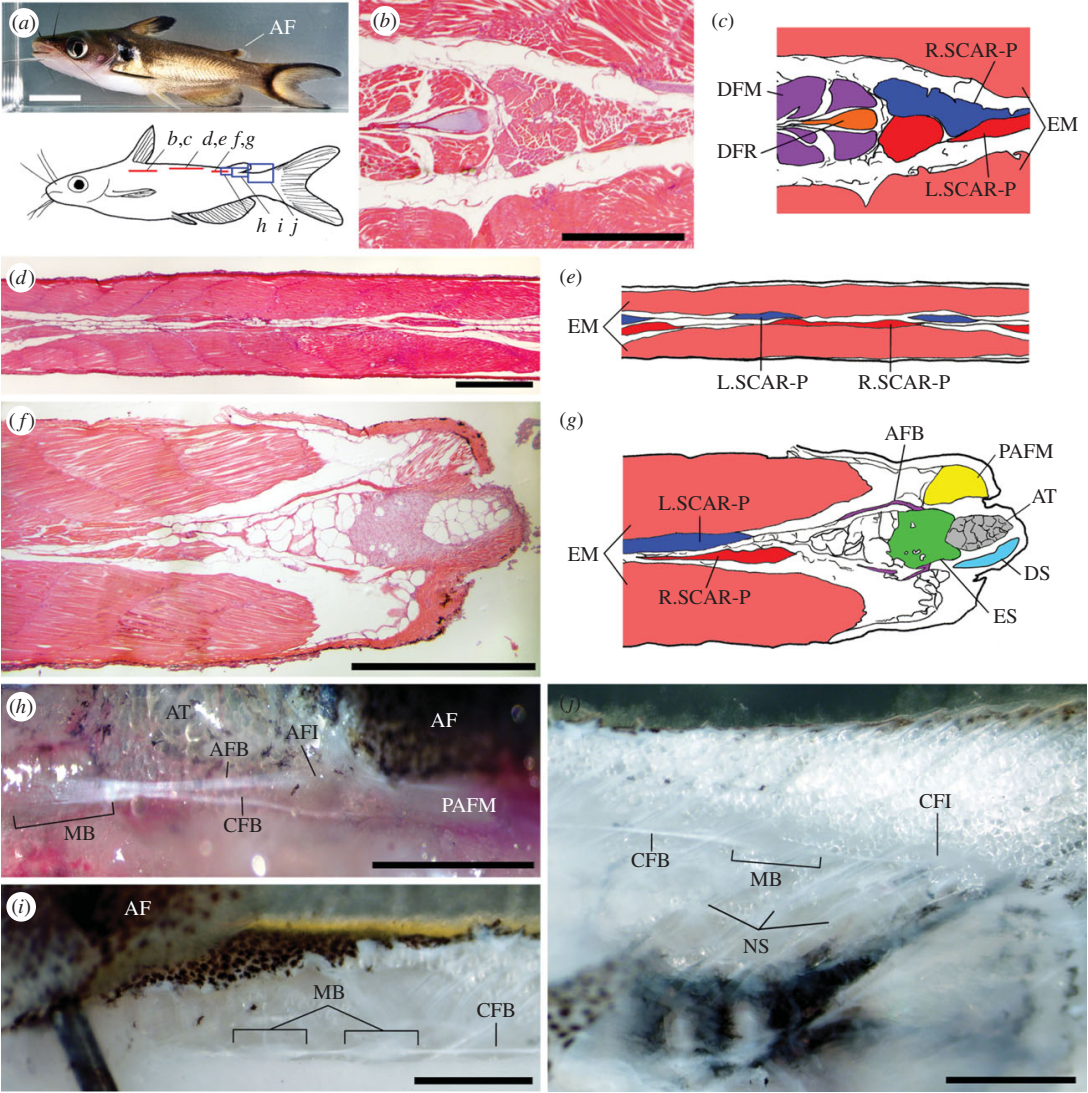
The SCAR-P inserts on both the adipose and caudal fins. (*a*) Photo of *H. brachysoma* indicating adipose fin (AF) (scale bar, 1 cm) and an illustration indicating the locations of sections (red lines) and dissections (blue rectangles) in subsequent panels. Photo credit Yen-Chyi Liu. Anterior is left in all panels. Sectioned materials (*b*,*d,f)* and their associated illustrations (*c,e,g*, respectively), are from the dorsal perspective; dissections (*h*–*j*) are viewed laterally and of the left side. (*b*,*c*) SCAR-P originates at the posterior-most radial of the dorsal fin. DFM, dorsal fin musculature; DFR, dorsal fin radial; EM, epaxial musculature; L.SCAR-P, left SCAR-P; R.SCAR-P, right SCAR-P. (*d*,*e*) The SCAR-P is organized as muscle bundles asymmetrically and serially arranged with tendons between the muscle bodies. (*f*,*g*) Insertion of the SCAR-P upon the adipose fin. The SCAR-P bifurcates along a tendinous region, in this case posterior to the muscle bundles, and the adipose fin branch (AFB) of the SCAR-P attaches laterally to an endoskeletal element (ES) at the posterior margin of the adipose fin. Immediately posterior to this attachment point the posterior adipose fin muscles (PAFM) attach latero-posteriorly upon the fin. The core of the fin is composed of adipose tissue (AT), and laterally supported by the dermal skeleton (DS), actinotrichia. (*h*) Bifurcation of the SCAR-P immediately anterior to the adipose fin insertion point. Here, as in most specimens, the AFB is composed of only tendon. The caudal fin branch (CFB) of the SCAR-P passes medially beneath the adipose fin. In this photo the CFB is obscured posteriorly by the PAFM, which inserts immediately posterior to the AFB's insertion point (AFI). MB, muscle bundle of the SCAR-P. (*i*) Immediately posterior to the adipose fin the SCAR-P muscle bodies of the CFB of the SCAR-P. (*j*) Caudal fin insertion (CFI) of the CFB of the SCAR-P. The SCAR-P inserts upon the distal tip of the neural spine of the posterior-most vertebrae. All scale bars, 1 mm.

**Figure 2.**
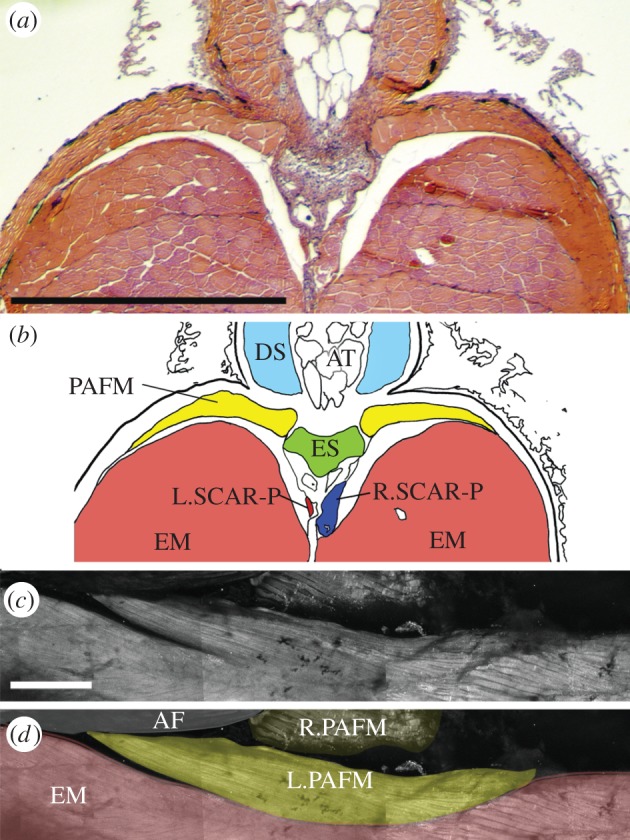
Posterior adipose fin muscles insert upon the adipose fin. (*a*) Transverse section and associated (*b*) illustration of the adipose fin immediately anterior to the posterior-most part of the adipose fin base. PAFMs are dorsal to epaxial musculature and thin further away from the insertion point upon the endoskeletal element (ES). DS, dermal skeleton; L.SCAR-P, left SCAR-P; R.SCAR-P, right SCAR-P. (*c,d*) Photograph and associated schematic of dissected tissue with skin removed that has been antibody stained for muscle, taken from a lateral and slightly dorsal perspective; anterior is to the left. AF, adipose fin; EM, epaxial musculature; L.PAFM, left PAFM; R.PAFM, right PAFM. All scale bars, 0.5 mm.

In most teleostean fishes, the SCAR-P muscles originate at the posterior-most radial of the dorsal fin and terminate upon the epurals, procurrent fin rays or the last neural spine anterior to the caudal complex [22]. Usually, the SCAR-P is continuous along its length and bilaterally symmetrical. In fishes with adipose fins, the SCAR-P generally reduces to a tendon beneath the adipose fin, dividing the muscle into anterior and posterior muscular subunits [22]. In *H. brachysoma*, the SCAR-P originates similarly, and at its origin the left and right sides of the SCAR-P are approximately symmetrical (figure 1*b,c*). Posteriorly, however, the muscles narrow both dorsoventrally and laterally, and the left and right sides develop asymmetry in their thickness, becoming alternatingly thicker and thinner as the muscles progress caudally. The degree of asymmetry increases until the SCAR-P is organized as a series of discrete muscle bundles interspersed by tendon (figure 1*d,e*). Immediately anterior to the posterior-most part of the adipose fin base, the SCAR-P tendon bifurcates, and a branch extends into the adipose fin. In most specimens (13 of 14 dissected), the adipose fin branch (AFB) of the SCAR-P was composed of only tendon; however, in one specimen a muscle bundle was observed along the AFB. At the base of the adipose fin is an endoskeletal element, upon which the AFB terminates (figure 1*f,g*). In coronal sections, the element is roughly oval at its dorsal-most extent (figure 1*f,g*), narrowing medially as it projects ventrally and inserts into the axial musculature (figure 2*a,b*). The cellular structure of the endoskeletal element is discussed below. The caudal fin branch of the SCAR-P passes medially beneath the adipose fin and inserts upon the distal tip of the neural spine of the posterior-most complete vertebra (figure 1*h–j*). Posterior to the adipose fin most of the length of the SCAR-P is tendinous, as muscles bundles become progressively more widely spaced (figure 1*i,j*). SCAR-P muscle bundles vary in their size, position and number both between the left and right sides of an individual (figure 1*d,e*) and among individuals (see the electronic supplementary material, table S1). The muscle fibres of the muscle bundles are oriented in parallel to one another and to the tendon.

### (b) A pair of muscles insert upon the adipose fin immediately posterior to the attachment site of the adipose fin branch of the SCAR-P

Immediately posterior to the SCAR-P tendon attachment a pair of muscles, which we named the posterior adipose fin muscles (PAFMs), insert bilaterally upon the endoskeletal element of the adipose fin (figures 1*f–h* and 2). The PAFMs overlay the epaxial musculature (figure 2*a,b*), and extend caudally from the insertion point. At their posterior end, the PAFMs are tightly affixed to the epaxial musculature and appear to originate from the fascia of the underlying epaxial musculature. The medial edges of the muscle meet at the midline immediately posterior to the adipose fin base. The lateral edges of the muscle extend laterally and wrap ventral-ward at an angle of approximately 45° (figure 2*c*). The muscles are very thin, narrowing further with distance from the muscle insertion (figure 2*a,b*). Because of this, the precise size of these muscles is difficult to assess by dissection. Antibody staining indicates that the PAFM extends posteriorly to a length approximately equal to the free margin of the adipose fin (figure 2*c*). The lateral extent of the muscle reduces more caudally. The PAFM fibres converge towards the point of insertion on the adipose fin (figure 2*c*).

### (c) Description of adipose fin ultrastructure

**Figure 3.**
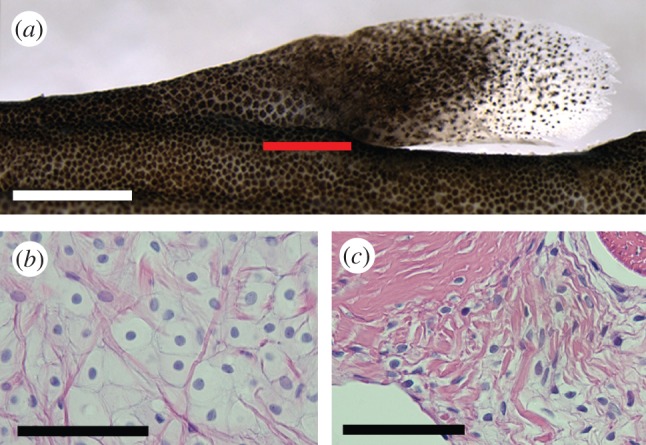
(*a*) The adipose fin of *H. brachysoma.* The red line indicates the position of sectioned material in subsequent panels. Scale bar, 1 mm. (*b*) Cells from the centre of the adipose fin endoskeletal element. The cells are disorganized and of variable size. (*c*) Endoskeletal cells (lower right) blend with dermal cells (upper left) at the dorsal margin of the element. Scale bars, 0.05 mm.

The adipose fin of *H. brachysoma* is superficially similar to those of salmonids, with the free portion of the adipose fin being approximately twice as long as it is tall (see figure 3*a* and electronic supplementary material, table S1). Actinotrichia, oriented proximo-distally in the fin membrane, support the fin (figure 2*a,b*), as in the adipose fins of other fishes [13]. The core of the fin is composed of adipose tissue (figures 1*f,g* and 2*a,b*), similar to that of *Ictalurus melas* [26]. The endoskeletal element did not stain with alizarin red or alcian blue. However, it shares key histological features with cartilage, and appears to be cartilage-related tissue (figure 3). Endoskeletal cells are of variable size and disorganized (figure 3*b*), similar to notochordal cartilage [27] and at its dorso-lateral margins the cells blend with dermal cells in a manner similar to fibrocartilage (figure 3*c*) [28].

### (d) Exploring function of the SCAR-P linkage in the adipose fin

**Figure 4.**
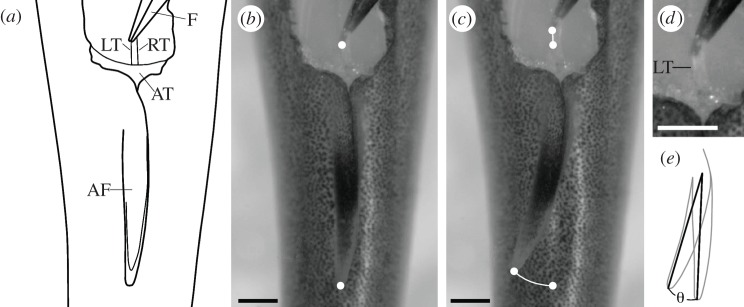
SCAR-P provides a putative mechanism for controlling adipose fin position. Photos and illustrations from the dorsal perspective, anterior is up. (*a*) A schematic of the dissection and manipulation used to test SCAR-P function. An anaesthetized fish was dissected and forceps (F) were used to pull one of the tendons anteriorly simulating unilateral SCAR-P contraction. AT, adipose tissue; LT and RT, left and right tendons, respectively, along the AFB of the SCAR-P. (*b*) Adipose fin at resting position with forceps holding the LT. (*c*) The LT is pulled anteriorly 0.3 mm, resulting in a leftward movement of the fin. (*d*) Close up of the forceps and tendon. (*e*) Angle of rotation (θ) was calculated by displacement of the adipose fin tip relative to the middle of the posterior point of the adipose fin that is attached to the body wall. Pulling the LT anteriorly by 0.3 mm results in a rotation of approximately 12°. Scale bars, 1 mm.

The SCAR-P has been explored in bluegill sunfish, *Lepomis macrochirus*, and found to be contracted unilaterally during swimming manoeuvres [29,30]. To simulate the effect of unilateral contractions of the SCAR-P on adipose fin kinematics, tendons of the AFB of the SCAR-P were unilaterally pulled anteriorly with forceps in one individual. Using the conservative estimate that the distance between origin of the SCAR-P and its insertion upon the adipose fin as 50 per cent muscle and 50 per cent tendon, the AFB of the SCAR-P tendon of one individual was pulled rostrally 0.3 mm, representing a 0.35 per cent contraction of the SCAR-P, easily within the bounds of muscle contractions during steady swimming in fishes [31]. Pulling the left AFB of the SCAR-P anteriorly by approximately 0.3 mm produced a marked leftward movement of the adipose fin, rotating the fin by 12° as measured from the middle of the posterior attachment point of the adipose-fin base to the fin tip (figure 4).

## 4. Discussion

Lineages have repeatedly traversed the functional discontinuity between primitively passive fins and a derived, actively controlled condition; how this is achieved remains poorly understood. Adipose fins are primitively passive structures. The musculoskeletal linkage system we identify in *H. brachysoma* is an innovation that reflects a gained potential to actively control fin movement and is the first such mechanism to be described in an adipose-fin system. This unique anatomy expands our understanding of the repeated process of musculoskeletal innovation in vertebrate fins. These data complement the palaeontological record and permit specific hypotheses to be proposed about the homology of the constituent parts, their phylogenetic order of acquisition, and the selective forces that might have promoted the origination of active control in this appendage.

**Figure 5.**
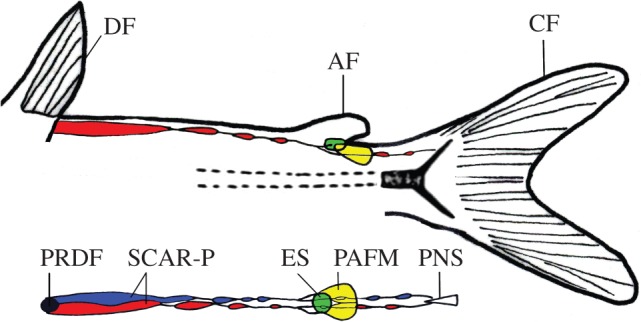
Summary of the musculoskeletal linkage of the adipose fin, not to scale. Top is from the lateral perspective, bottom is dorsal. AF, adipose fin; CF, caudal fin; DF, dorsal fin; ES, endoskeletal element of the adipose fin; PNS, posterior-most neural spine; PRDF, posterior radial of the dorsal fin; SCAR-P, supracarinalis posterior; PAFM, posterior adipose fin muscle.

The novel musculoskeletal linkage system observed in the adipose fin of *H. brachysoma* includes two sets of muscles and an endoskeletal element (figure 5). The first muscle, the SCAR-P, became associated with the adipose fin through the co-option of an existing functional system, bifurcating a primitively linear musculoskeletal linkage. The second muscle, the PAFM, is of uncertain homology, though its shape and position suggest origination by subdivision of the dorsal-most region of a myomere. There is precedence for such an origin; the intrinsic musculature of actinopterygian caudal fins is hypothesized to have arisen similarly by the subdivision and subfunctionalization of epaxial musculature [22,32,33]. The anatomy of the PAFM is reminiscent of dorsal inclinator (DI) muscles in median fins [22], and it is possible that the origination of these de novo muscles involved the co-option of developmental modules from other fin-associated musculature, such as the DI. The endoskeletal element observed in the base of the adipose fin in *H. brachysoma* is the first to be identified among fishes with adipose fins that originated within the Otophysi. The cartilage-like structure observed here is, therefore, convergent with the endoskeletal elements previously described in other adipose fins. It is unclear whether such elements generally are adaptive, serving to stiffen or support the fin, or whether they are simply a consequence of mechanical loading and compressive forces exerted upon the fin promoting the development of cartilage-related tissues [34,35].

Neither an endoskeleton nor associated muscles have been identified in fishes closely related to *H. brachysoma*, prohibiting conclusions regarding the relative order of acquisition of these linkage components. However, given that cartilage has repeatedly developed at the base of adipose fins, we propose that the endoskeletal element arose first and that muscular associations evolved secondarily, allowing the cartilage-like tissue to provide a substrate for muscular attachment.

Patterns of increasing anatomical complexity and evidence for the evolution of new functional systems in fins have emerged from palaeontological data of early vertebrates. For example, pectoral fins originated as paired dermal structures in extinct jawless fishes [1,2,36]. While some have suggested these structures might have been controlled independently of axial musculature [37], the poor preservation of soft tissues and a lack of endoskeleton confound these hypotheses. In the lineage leading to gnathostomes pectoral fins were elaborated upon. *Escuminaspis laticeps*, an osteostracan (the group sister to the earliest vertebrates with jaws) with a monobasal pectoral fin endoskeleton and endoskeletal girdle with sites for muscular attachment points, branchial nerves and vascularization, provides the first evidence for active control of these appendages [38]. Our data show that the morphological changes underlying the functional transformation of an appendage, from primitively passive to actively controlled, can involve subtle re-organizations of the soft-tissue anatomy. It is likely that such changes would leave no hard-tissue signature that would be detectable in fossil remains. Therefore, hypotheses of the evolution of function in novel appendages based upon records of hard-tissue anatomy will tend towards the conservative.

In *H. brachysoma,* the SCAR-P's organization as a series of discrete asymmetrically arranged muscle bodies is unique and functionally intriguing. We are unaware of analogous organizations of muscle in other musculoskeletal systems. This morphology would seem to imply a degree of coordination or concerted contraction among the muscular subunits, as the independent contraction of a particular muscle body would likely result in the stretching of adjacent muscle bodies along the series with little effectual result towards ultimately moving the structures upon which the SCAR-P inserts. In fishes with adipose fins, the SCAR-P is reduced to a tendon as it passes beneath the adipose fin [22]. This lends support to the hypothesis that such discrete muscle bodies might arise by the intermittent reduction of muscle along a primitively continuous SCAR-P, rather than by the addition of muscle along regions of the SCAR-P that were once tendinous. The functional implications of the asymmetry in the SCAR-P of *H. brachysoma* are unclear. The specimens used in this study were adults, though not full sized, and it is possible that the asymmetry and distribution of muscle along the SCAR-P is a function of growth. It would be interesting to examine whether SCAR-P morphology varies over ontogeny.

The musculoskeletal linkage presented here reflects a previously undescribed functional potential for adipose fins. The SCAR-P inserts upon both the adipose and caudal fins, indicating an integration of their kinematics. Previous studies of SCAR-P function in other species have found that this muscle is recruited unilaterally to raise the dorsal portion of the caudal fin during steady swimming [29]. Our simulations of unilateral contractions of the SCAR-P suggest that this muscle can deflect the adipose fin laterally. This could serve a function analogous to the second dorsal fins of some chondrithyans, which are actively controlled to direct flow towards the caudal fin, thereby augmenting thrust production [39]. Elucidating the function of this linkage will require physiological studies such as muscle stimulation and electromyographs to differentiate between passive and active fin movements during swimming. Additionally, digital particle image velocimetry provides an avenue for discovering how adipose fins affect flow broadly and would inform understanding of adipose fin function.

Adipose fins are morphologically diverse, varying in tissue composition, shape and position [9,40–42]. However, it is only recently that they have been regarded as adaptive structures that potentially serve a variety of functions, which, as of yet, remain poorly understood. Hypotheses of these functions include interspecific signalling [14] and hydrodynamic effects limited to larval stages [43]. Mounting evidence indicates, however, that adipose fins function largely to facilitate high-performance swimming [44,45]. The mechanism by which these fins might garner such a performance advantage is unclear, but it has been proposed that this is achieved by affecting pre-caudal flow to augment thrust production or serving as a sensory structure [13,44,46]. And while we cannot discriminate whether the innovation in *H. brachysoma* reflects the evolution of a new function or specialization of a pre-existing function [47], we propose that, in either case, the ability to modulate adipose fin position indicates adaptation. As adipose fins are found in speciose and morphologically diverse groups, it is possible that analogous musculoskeletal linkages have evolved in other species. Further comparative reviews may give insight into how novel appendages are elaborated upon evolutionarily to produce complex musculoskeletal systems.
